# Microfluidics-engineered ovalbumin-carvacrol nanoparticles for ultrasonically fabricated pickering emulsion coatings: a sonochemical strategy for fruit preservation^☆^

**DOI:** 10.1016/j.ultsonch.2026.108073

**Published:** 2026-09-23

**Authors:** Song-Wei Lv, Zhu-Huan Tang, Zhi-Fan Ye, Yi-Jing Lu, Gao-Xiang Jin, He-Ling Yu, Da-Na Liu, Jianhao Wang

**Affiliations:** School of Pharmacy &·Affliated Hospital of-Changzhou University (Changzhou West TaihuHospital), Changzhou University, Changzhou 213164, China

**Keywords:** Microfluidics, Ultrasound emulsification, Acoustic cavitation, Pickering emulsion, Edible composite film, Postharvest preservation, Passion fruit

## Abstract

Postharvest spoilage of fresh fruits causes substantial global food loss and exacerbates environmental burdens associated with conventional plastic packaging, driving the urgent need for sustainable and effective preservation alternatives. Herein, we address this challenge through a microfluidics–ultrasound route that fabricates edible chitosan coatings reinforced with ovalbumin (OVA) Pickering emulsions for passion fruit preservation. Microfluidic precision synthesis enabled the production of uniform OVA-carvacrol (Car) nanoparticles with narrow size distribution and excellent batch-to-batch reproducibility-advantages not achievable by conventional bulk mixing. Subsequently, ultrasonic emulsification (20 kHz, 100–400 W, 2–8 min) was applied, and the acoustic cavitation effects were systematically exploited to refine emulsion droplet dimensions, reinforce the interfacial adsorption of OVA-Car nanoparticles, and tailor the viscoelastic properties of the Pickering emulsion network. The optimally ultrasonicated emulsion (300 W, 6 min) exhibited significantly enhanced rheological performance and sustained release behavior. When compounded with chitosan to form edible coatings, the composite film delivered potent synergistic antioxidant and antimicrobial activities, with DPPH and ABTS radical scavenging rates of 66.92 % ± 1.48 % and 91.40 % ± 0.29 %, respectively, and ≥ 75 % inhibition against *S. aureus*, *E. coli*, and *A. niger*. Coating application restricted passion fruit weight loss to 10.2 % (vs. 17.4 % in uncoated controls) and extended shelf life to 7 days at ambient temperature. This work demonstrates that the integration of microfluidic nanoparticle engineering with ultrasonic cavitation modulation offers a scalable, clean-label sonochemical strategy to supplant synthetic preservatives and petroleum-derived packaging in postharvest fruit preservation.

## Graphical abstract

**Figure:**
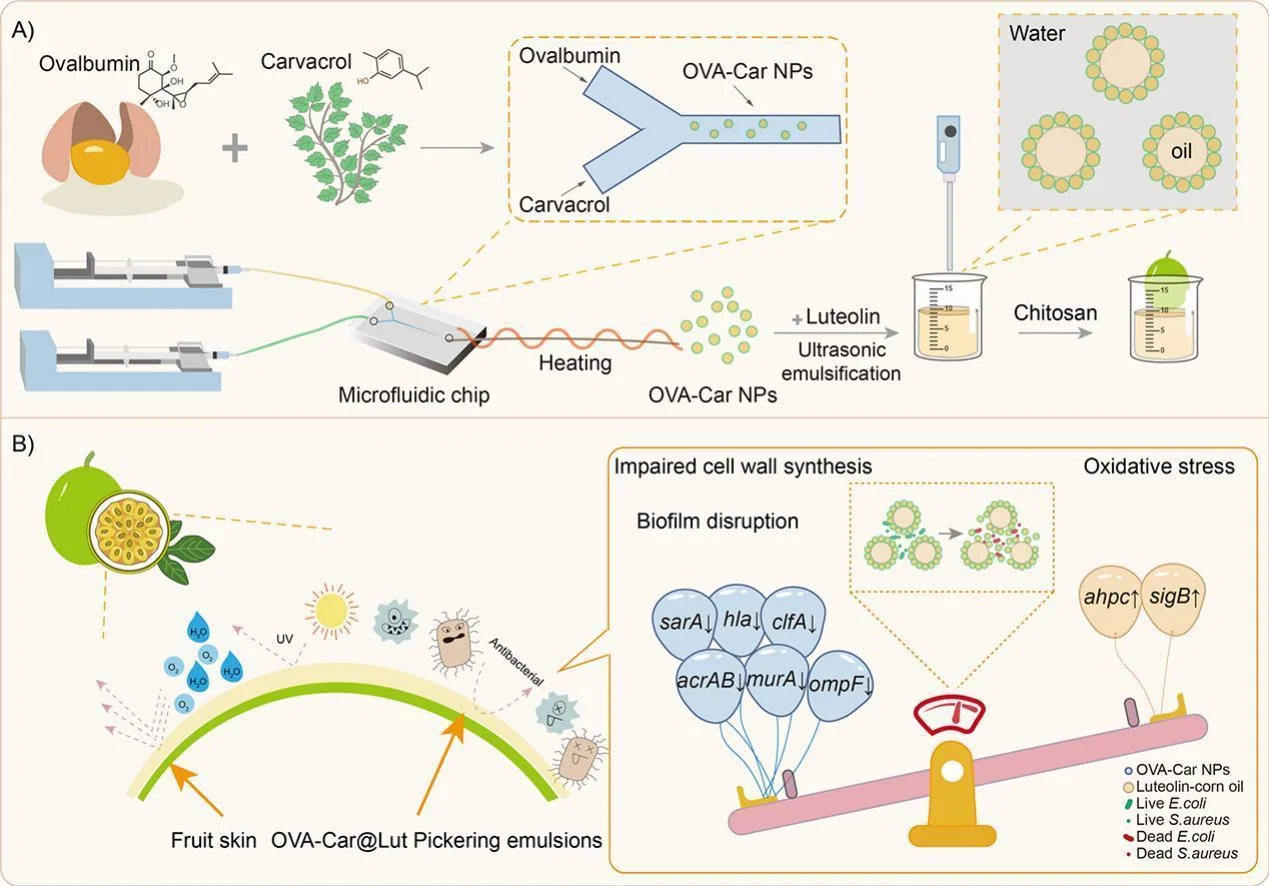

## 1 Introduction

Fresh fruits and vegetables are rich in dietary nutrients and bioactive phytochemicals, forming an indispensable component of balanced human diets [1]. Nevertheless, fresh horticultural products suffer from drastically shortened postharvest shelf lives triggered by three dominant factors: pathogenic microbial contamination, continuous physiological water loss, and endogenous metabolic aging [2], [3]. Globally, ∼30 % of fresh produce is lost postharvest, causing significant economic and resource pressures [3].

Traditional postharvest preservation approaches including cold storage, controlled atmosphere (CA) treatment, and synthetic chemical preservatives are widely adopted in commercial production, yet each suffers from inherent limitations [4]. Cold storage requires massive energy consumption; CA systems rely on costly, complicated supporting equipment; synthetic preservatives raise latent toxic risks to human bodies and ecological environments [5]. Moreover, disposable petroleum-derived plastic packaging aggravates solid waste pollution and risks of additive leaching.

This has driven ongoing research into sustainable, low-cost preservation alternatives suitable for food applications [6]. Among various functional materials, Pickering emulsion-stabilized edible biopolymer coatings have gained considerable research attention. The selection of particulate stabilizers exerts a critical influence on emulsion performance. A broad range of building blocks, including inorganic nanoparticles, protein nanoparticles, polysaccharide particles, and nanogels, have been widely explored for emulsion stabilization. Nanogels are particularly attractive owing to their softness, deformability, and stimuli-responsive properties, allowing dynamic adaptation at the oil-water interface. For example, Cui and co-workers reported that amphiphilic nanogels with tailored hydrophobicity are capable of stabilizing both oil-in-water and water-in-oil emulsions, and constructed a quantitative framework for predicting phase inversion from polymer-solvent interactions [7]. Furthermore, nanogel-stabilized Pickering droplets can be used to fabricate colloidosomes, representing a promising strategy for encapsulation and controlled release [8]. Inspired by these previous findings on versatile particulate stabilizers, we chose ovalbumin as our food-grade protein building block. Unlike conventional systems relying on chemical surfactants, these coatings are stabilized by food-grade solid particles [6], [9], [10]. They exhibit superior structural stability, full biocompatibility, and high loading efficiency for lipophilic bioactive compounds. When engineered into edible films and coatings, Pickering emulsions form a dense protective barrier on fruit surfaces, which suppresses water transpiration, inhibits microbial adhesion, and mitigates lipid and enzymatic oxidation. This synergistic protective effect effectively alleviates postharvest quality deterioration of fruits and vegetables [11], [12]. Nevertheless, conventional batch fabrication of biopolymer stabilizers suffers from inherent technical drawbacks, including heterogeneous particle size distribution and poor batch-to-batch reproducibility. These bottlenecks severely limit the industrial translation of Pickering emulsion coating technologies, highlighting the necessity of advanced physical processing strategies for performance improvement [13], [14].

The physicochemical fabrication protocols of stabilizing particles (particle size, polydispersity, surface hydrophobicity, interfacial adsorption affinity) directly determine the final quality of Pickering emulsions, which is highly dependent on particle fabrication protocols [15], [16]. Traditional nanoparticle preparation approaches, such as solvent evaporation and batch emulsion polymerization, typically yield particles with broad size distributions and low preparation repeatability, restricting their scalable industrial implementation [17]. As an emerging precision fluid processing technique, microfluidics enables fine-tuning of multiphase mixing and mass transfer at micrometer scales, which has increasingly been utilized to fabricate food-grade biopolymer nanoparticles [18], [19], [20]. Microfluidic synthesis offers distinct technical advantages, including tunable particle dimensions, narrow size polydispersity, excellent batch consistency, and great compatibility with continuous manufacturing workflows. While microfluidics enables the generation of highly uniform droplets, its use for emulsification is hindered by low throughput, intricate chip fabrication, and their poor industrial scalability-key bottlenecks prevalent in Pickering emulsion research. Ultrasonic emulsification effectively overcomes these drawbacks by virtue of its high energy efficiency and scalable continuous operation. Moreover, acoustic cavitation can actively promote particle assembly at the oil–water interface and facilitate the formation of robust interfacial networks. Accordingly, this work adopts a two-stage hybrid strategy: microfluidics is employed for the controllable synthesis of nanoparticles, while ultrasonication serves for high-throughput Pickering emulsion fabrication. This integrated approach combines the precision of microfluidics with the productivity of ultrasonication, providing a feasible pathway for the industrial translation of Pickering emulsion technology [21], [22]. Notably, the synergistic combination of microfluidic-fabricated protein nanoparticles with ultrasonic emulsification for the development of postharvest preservation Pickering coatings remains underexplored [23]. As a powerful sonochemical intensification strategy, ultrasonic treatment induces acoustic cavitation to modulate particle interfacial characteristics, refine emulsion droplet morphology, and build compact interfacial networks. Such sonochemical modulation significantly enhances the interfacial stability and functional performance of microfluidic-derived Pickering emulsion systems [24], [25].

Antibacterial and antioxidant performances are essential functional properties that determine the practical efficacy of edible Pickering emulsion coatings. Carvacrol (Car), a natural plant-derived polyphenol, exhibits excellent broad-spectrum bacteriostatic performance and high free radical scavenging ability. However, its inherent poor water solubility restricts its direct incorporation into aqueous coating matrices. Ovalbumin (OVA), the predominant protein in egg white, is a cheap, readily available, amphipathic food-grade raw material suitable for fabricating Pickering stabilizers [26]. Mild thermal denaturation can unfold OVA molecular chains and expose internal hydrophobic residues, greatly boosting its interfacial adsorption capacity and enabling efficient encapsulation of lipophilic carvacrol [27], [28], [29]. Nevertheless, systematic fabrication of OVA-carvacrol composite nanoparticles via precise microfluidic synthesis coupled with ultrasonic emulsification for fresh fruit postharvest preservation remains rarely explored. To further improve the oxidative stability of the coating system, lipophilic luteolin, a natural flavonoid with strong antioxidant activity, was incorporated into the oil phase to complement the antimicrobial function of carvacrol. This combination may yield synergistic antimicrobial‑antioxidant preservation effects, which can counteract both microbial spoilage and oxidative deterioration of fruit.

This work develops a novel microfluidic-ultrasonic coupled fabrication strategy to construct high-performance edible Pickering emulsion coatings for passion fruit preservation, addressing the poor batch uniformity and limited functional efficiency of conventional natural preservation systems. A two-inlet single-outlet microfluidic chip is first adopted to synthesize uniform heat-denatured OVA-carvacrol nanoparticles as solid Pickering stabilizers. Subsequently, gradient ultrasonic power and duration treatments are applied to prepare optimized Pickering emulsions, and the as-obtained emulsions are compounded with chitosan (a film-forming matrix) to form integrated edible coatings. Results demonstrate that microfluidic precision synthesis effectively improves the dimensional uniformity and batch-to-batch reproducibility of OVA-carvacrol nanoparticles, while controllable ultrasonic cavitation further modulates particle interfacial adsorption, optimizes emulsion rheological behaviors, and tailors the sustained release profile of encapsulated carvacrol. The synergistic antibacterial and antioxidant preservation effects of the composite edible coating are fully characterized using postharvest passion fruit as the research object. Overall, this study establishes a green and scalable sonochemical processing platform for the fabrication of high-performance edible coating materials, offering feasible technical support for the shelf-life extension of fresh horticultural products.

## 2 Materials and methods

### 2.1 Materials

Ovalbumin (OVA, ≥98 %), carvacrol (Car, ≥99.0 %), 1,1,3,3-tetramethoxypropane, thiobarbituric acid (≥98.0 %), and chitosan (deacetylation degree ≥95.0 %) were sourced from Adamas-beta (Shanghai, China). Luteolin (Lut, ≥98 %) was purchased from Shanghai Aladdin Biochemical Technology Co., Ltd. (Shanghai, China). DPPH colorimetric assay kit and Folin–Ciocalteu phenol reagent were obtained from Yuanye Bio-Technology (Shanghai, China). Live/dead bacterial viability kit was purchased from Thermo Fisher Scientific (Waltham, MA, USA). Phosphate-buffered saline (PBS, 0.01 mol/L, pH 7.4), dialysis bags (MWCO = 3500 Da), and Milli-Q deionized water (18.2 MΩ·cm) were used throughout all experiments.

### 2.2 Preparation and characterization of OVA-Car NPs

#### 2.2.1 Preparation protocols

A 15 mg/mL OVA solution was prepared by dissolving 150 mg OVA in 10 mL PBS (25 ℃, 500 rpm, 30 min). A 3 mg/mL Car ethanol solution was formulated by dissolving 15 mg Car in 5 mL anhydrous ethanol, followed by ultrasonic treatment (40 kHz, 100 W, 5 min).

##### 2.2.1.1 Microfluidic fabrication

A Y-shaped glass microfluidic chip (channel width 200 μm, depth 100 μm) connected to dual precision syringe pumps was used for continuous synthesis of OVA-Car NPs. The aqueous OVA phase and the carvacrol ethanol phase were separately pumped into the chip. Two key parameters were optimized: the water/oil flow rate ratio (1:1, 2:1, 3:1, 4:1, 5:1, v/v) and the total flow rate (3, 4, 5, 6, 7 mL/min) [30]. The collected nanoparticle suspension was stored at 4 °C for subsequent experiments.

##### 2.2.1.2 Conventional dropwise injection

For comparison, 1 mL of carvacrol ethanol solution was rapidly injected into 5 mL of OVA solution under magnetic stirring at 1500 rpm and 25 °C. The mixed system was then heated in a 90 °C water bath to induce thermal denaturation of OVA, followed by natural cooling to room temperature to obtain conventional OVA-Car nanoparticles, which were stored at 4 °C.

#### 2.2.2 Characterization of OVA-Car NPs

Dynamic light scattering (DLS, Malvern Zetasizer Nano) was employed to measure the hydrodynamic particle size and polydispersity index (PDI) of OVA-Car NPs. Fourier-transform infrared (FT-IR) spectra (Nicolet iS50) were recorded for pure OVA, pure carvacrol, and freeze-dried OVA-Car NPs over the wavenumber range of 4000–400 cm^−1^. Intrinsic fluorescence spectra (Edinburgh HAI-5H1-0004) were acquired at an excitation wavelength of 280 nm, with emission scanned from 300 to 400 nm and a slit width of 5 nm. The micromorphology of freeze-dried nanoparticles was observed via scanning electron microscopy (SEM, FEI Nova450) after gold sputtering. The surface hydrophobicity of nanoparticle powder was characterized using a contact angle tester (Kruss FM4000): compressed nanoparticle tablets were prepared, distilled water droplets were deposited on the surface, and equilibrium contact angles were recorded.

### 2.3 Fabrication of OVA-Car stabilized Pickering emulsions

#### 2.3.1 Emulsion preparation procedure

Corn oil was used as the oil phase. For luteolin‑loaded formulations, Lut was dissolved in corn oil by magnetic stirring at 40 °C for 30 min to achieve full dissolution. The final concentration of Lut in the emulsion was in the range of 13.125–105 μg/mL, which was the concentration condition adopted for the DPPH and ABTS free radical scavenging assays. To optimize the emulsion formulation, Pickering emulsions were prepared with water/oil volume ratios ranging from 3:7 to 7:3 (v/v), while keeping the nanoparticle concentration constant. The emulsions were characterized by droplet size, stability index, and creaming behavior after 7 days of storage. Based on the smallest droplet size and highest stability, an oil phase volume fraction of φ = 0.7 (water/oil = 3:7, v/v) was selected as optimal and used for all subsequent experiments. The optimized microfluidic OVA-Car nanoparticle aqueous solution was mixed with the Lut-containing corn oil and pre-emulsified using a high-speed homogenizer at 15,000 rpm for 3 min to obtain a crude emulsion.

Subsequently, the crude emulsion was subjected to ultrasonic treatment using a 20 kHz probe sonicator equipped with a Φ6 mm titanium alloy probe. [31]. The amplitude was set at 60 % of the maximum amplitude (corresponding to 60 μm peak-to-peak, based on a maximum amplitude of 100 μm for the Φ6 mm probe), with the ultrasonic power set at varying nominal outputs (100, 200, 300, and 400 W) as indicated on the instrument. The treatment was performed in pulse mode (5 s on / 5 s off) with ice-bath cooling to prevent thermal degradation of biopolymers, and a fixed treatment volume of 30 mL per batch. Two ultrasonic variables were systematically varied: ultrasonic power gradient: 100, 200, 300, 400 W (with sonication time fixed at 6 min); ultrasonic time gradient: 2, 4, 6, 8 min (with power fixed at 300 W).

#### 2.3.2 Comprehensive characterization of Pickering emulsions

##### 2.3.2.1 Microscopic morphological observation

Optical microscopy: Emulsion samples were diluted 5-fold with deionized water to reduce droplet overlap. An aliquot (20 μL) of the diluted emulsion was dropped onto a glass slide and covered with a coverslip. Droplet images were captured using a Nikon ECLIPSE Ti2 inverted microscope, and droplet size distribution was analyzed using ImageJ software.

Confocal laser scanning microscopy (CLSM): Oil droplets were stained with 0.1 wt % Nile Red (lipophilic dye), and OVA-Car nanoparticles were labeled with 0.1 wt % FITC. A 10 μL aliquot of stained emulsion was prepared for observation to visually assess the thickness and integrity of the particle interfacial adsorption layer on oil droplets.

##### 2.3.2.2 Rheological characterization

Rheological measurements were performed using a NETZSCH KINEXUS rotational rheometer at 25 °C with a parallel plate fixture (gap 1 mm). Dynamic strain sweep was first conducted to determine the linear viscoelastic region (LVR). Frequency sweep (0.1–100 rad/s) was then performed to record the storage modulus (G′) and loss modulus (G″). Steady-state flow measurements (shear rate: 0.1–100 s^−^1) were carried out to determine the apparent viscosity of emulsions under different ultrasonic conditions. All rheological tests were performed in triplicate.

##### 2.3.2.3 In vitro sustained release behavior

An aliquot (2 mL) of fresh emulsion was loaded into a dialysis bag (MWCO 3500 Da) and immersed in 35 mL of release medium (PBS pH 5.5 containing 20 % v/v ethanol) under gentle shaking at room temperature. At predetermined time points, 2 mL of release medium was withdrawn for UV–vis detection at 278 nm (the characteristic absorption wavelength of carvacrol), and an equal volume of fresh medium was replenished. The cumulative release rate of carvacrol was calculated based on standard calibration curves.

##### 2.3.2.4 Antioxidant capacity (DPPH and ABTS assays)

The antioxidant activities of Pickering emulsions containing different concentrations of luteolin (ranging from 13.125 to 105 μg/mL in the final emulsion, as prepared in Section 2.3.1) were evaluated using DPPH and ABTS radical scavenging assays.

DPPH assay: The emulsion diluent was mixed with 0.1 mM DPPH ethanol solution at a 1:1 (v/v) ratio, incubated in the dark for 30 min, and the absorbance at 517 nm was recorded.

ABTS assay: ABTS working solution was prepared by reacting 7 mM ABTS with 4.95 mM potassium persulfate for 12–16 h in the dark, then diluted with ethanol to an absorbance of 0.70 ± 0.02 at 734 nm. After mixing the emulsion with the working solution and incubating in the dark for 6 min, the absorbance at 734 nm was measured. Radical scavenging rates were calculated for all ultrasonic emulsion groups for comparison.

##### 2.3.2.5 Emulsion stability evaluation

Centrifugal stability: An aliquot (10 mL) of emulsion was centrifuged at 8000×g for 20 min, and the mass of the separated oil layer was weighed to calculate the oil precipitation rate.

Thermal stability: Emulsions were heated at 90 °C for 20 min, and the change in average droplet size was measured to evaluate thermal resistance.

Storage stability: Emulsions were stored at 4 °C for 28 days, and the creaming index (CI) was recorded at regular intervals.

### 2.4 Antibacterial activity of ultrasonic-treated Pickering emulsions

Test strains: Staphylococcus aureus (Gram-positive), Escherichia coli (Gram-negative), and Aspergillus niger (mold).

#### 2.4.1 Colony counting method

Bacterial suspension (10⁶ CFU/mL) was mixed with emulsion samples at a volume ratio of 1:3 and incubated at 37 °C for 1.5 h. Serial gradient dilution was performed, and 100 μL of the diluted solution was spread onto agar medium, cultured at 37 °C for 24 h, and the colony number was counted to calculate bacterial survival rate. The PBS-treated group served as the blank control.

#### 2.4.2 Live/dead bacterial CLSM observation

An aliquot (20 μL) of SYTO 9/PI live/dead fluorescent dye was added to the bacterial suspension treated with emulsions and incubated in the dark at 37 °C for 20 min. A 10 μL aliquot of the stained sample was prepared for CLSM observation to distinguish live and dead bacterial populations.

### 2.5 Antibacterial mechanism analysis of emulsions

#### 2.5.1 Bacterial micromorphology (SEM)

Logarithmic-phase bacteria were treated with emulsions at 37 °C for 1.5 h, then centrifuged at 4 °C to collect the cells, fixed with 4 wt % glutaraldehyde, dehydrated through a graded ethanol series (50 %, 70 %, 90 %, and 100 %), freeze-dried, and gold-sputtered for SEM observation of cell surface damage.

#### 2.5.2 Biofilm eradication assay

Aliquots (200 μL) of S. aureus or E. coli suspension were inoculated into 96-well plates and cultured at 37 °C for 48 h to form mature biofilms. The supernatant was discarded, emulsions were added, and the plates were incubated for another 1.5 h. The wells were washed twice with PBS, stained with crystal violet for 10 min, rinsed to remove excess dye, and the bound crystal violet was dissolved in 80 % ethanol. The absorbance at 600 nm was measured to quantify residual biofilm biomass.

#### 2.5.3 Biofilm formation inhibition assay

Bacterial suspension and emulsions were simultaneously added to 96-well plates and incubated at 37 °C for 48 h. The supernatant was discarded, and the crystal violet staining procedure was performed as described in Section 2.5.2. The absorbance at 590 nm was measured to evaluate biofilm formation inhibition efficiency.

#### 2.5.4 RT-qPCR gene expression analysis

Bacteria treated with PBS (control) and the optimal ultrasonic emulsion were collected after 1.5 h of incubation at 37 °C, centrifuged, and washed with PBS three times, then stored at -80 °C for RNA extraction and reverse transcription quantitative PCR detection. The related primer sequences are listed in Table S1.

### 2.6 Fabrication and characterization of chitosan–emulsion composite edible films

#### 2.6.1 Film preparation

A 2 % (w/w) chitosan solution was prepared by dissolving chitosan in 1 % acetic acid aqueous solution. Chitosan‑based Pickering emulsion composite films were fabricated by blending the optimized Pickering emulsion with chitosan solution at volume ratios (v/v) of 0:10, 1:9, 3:7, and 5:5. These ratios correspond to final Pickering emulsion fractions of 0  %, 10  %, 30  %, and 50  %, respectively. Each mixture was stirred at room temperature for 8 h to yield homogeneous film‑forming solutions. The resulting solutions were cast onto Petri dishes and dried under constant‑temperature conditions for 12 h to produce edible composite films. According to mechanical and barrier performance results (Section 3.3), the 50  % (v/v) formulation was chosen for subsequent passion‑fruit‑coating experiments.

#### 2.6.2 Film physical and barrier properties

##### 2.6.2.1 Light transmittance and surface contact angle

Film samples were cut into 2 cm × 2 cm squares. A UV-Vis spectrophotometer was used to measure light transmittance over the range of 200–600 nm. A contact angle goniometer was used to record the water contact angle on the film surface.

##### 2.6.2.2 Water vapor permeability (WVP)

Anhydrous calcium chloride (2 g) was placed in glass bottles (diameter: 2 cm), and the bottle openings were sealed with circular film samples (diameter: 2.5 cm) secured with adhesive tape. The sealed bottles were placed in a saturated NaCl environment for 12 h and weighed to record mass change. WVP was calculated using Eq. (1)

(1) $$\text{WVP=}\frac{\Delta \text{M}\times \text{d}}{\text{A}\times \text{T}\times \Delta \text{P}}$$

where ΔM is the mass change of the bottle (g), d is the film thickness (m), A is the effective permeation area (m^2^), T is the permeation time (h), and ΔP is the water vapor partial pressure difference across the film.

##### 2.6.2.3 Swelling ratio (SR)

Film samples (2 cm × 2 cm) were immersed in PBS for 24 h. Surface free water was removed with filter paper, and the samples were weighed. The swelling ratio was calculated using Eq. (2):

(2) $$\text{SR}=\frac{{\text{m}}_{2}\text{-}{\text{m}}_{\text{1}}}{{\text{m}}_{\text{1}}}\text{×100\%}$$

where m_1_ is the initial film mass (g), and m_2_ is the mass after swelling equilibrium (g).

### 2.7 Coating treatment and postharvest quality evaluation of passion fruits

Fresh passion fruits were cleaned with tap water and randomly divided into three groups: blank control (no coating), pure chitosan film group, and optimal ultrasonic emulsion–chitosan composite coating group, with 20 fruits per group. A dip-coating method was adopted (fruits were immersed in the coating liquid for 30 s, followed by natural air drying). All fruits were stored at 22 ± 2 °C, and the following indices were measured on days 0, 3, 5, and 7 (n ≥ 3 biological replicates): weight loss rate, fruit firmness, total soluble solids (TSS), titratable acidity (TA), vitamin C, and total phenol content.

#### 2.7.1 Volatile flavor compound analysis

Passion fruit pulp (20 g) was mixed with 40 mL of n-hexane/diethyl ether (2:1, v/v), homogenized for 2 min, and subjected to ice-bath magnetic stirring for 30 min, followed by centrifugation at 8000 rpm for 10 min. The supernatant was collected, concentrated by rotary evaporation and nitrogen blowing to remove the solvent, and then analyzed by GC-MS for volatile components.

#### 2.7.2 Peel micromorphology

Passion fruit peel samples at different storage time points were rapidly frozen with liquid nitrogen, freeze-dried, and observed by SEM to examine surface structural changes during senescence.

#### 2.7.3 Shrinkage index

The shrinkage scoring standard was defined as: 0 (no shrinkage) to 9 (over 80 % shrinkage), with each interval representing 10 %. The shrinkage index was calculated using Eq. (3):

(3) $$\text{Shrinkage}\;\;\text{index=}\frac{\sum \text{S}\text{×n}}{\text{N}}$$

where S is the shrinkage score of a single fruit, n is the number of fruits corresponding to that score, and N is the total number of fruits in each group.

### 2.8 Statistical analysis

All experiments were conducted with at least three technical replicates, and in vitro functional tests were repeated more than three times. Experimental data are expressed as mean ± standard deviation (SD). One-way analysis of variance (ANOVA) was used for multi-group comparison, and the independent Student’s t-test was used for two-group comparisons. GraphPad Prism 8 was used for all statistical calculations, and a P-value < 0.05 was considered statistically significant.

## 3 Results and discussion

### 3.1 Preparation and characterization of OVA-Car particles

A Y-shaped vortex microfluidic chip was designed for the fabrication of OVA-Car nanoparticles via an antisolvent precipitation method coupled with thermal treatment, enabling simultaneous particle formation and heat-induced structural modification (Fig. 1A). Sufficiently small particle dimensions are critical for Pickering emulsifiers to achieve tight interfacial adsorption at oil–water interfaces and build compact protective layers against droplet coalescence [32]. Fig. 1B and C illustrate optimization results for the microfluidic preparation parameters for OVA-Car NPs. With increasing aqueous-to-organic phase ratio and total flow rate, particle size and polydispersity index (PDI) of the fabricated NPs exhibited a gradual decreasing trend. The minimum particle size was obtained at an OVA solution to Car solution flow rate ratio of 5:1 and a total flow rate of 7 mL/min. Such rapid microscale mixing generates high supersaturation degrees, which facilitates fast nucleation while inhibiting excessive particle growth, thereby producing nanoparticles with reduced size and improved size homogeneity. Compared with the conventional injection method (Fig. 1D), microfluidic processing improves production efficiency and produces finer nanoparticles (129.6 nm vs. 184.1 nm) with narrower size distributions [30].

**Fig. 1:**
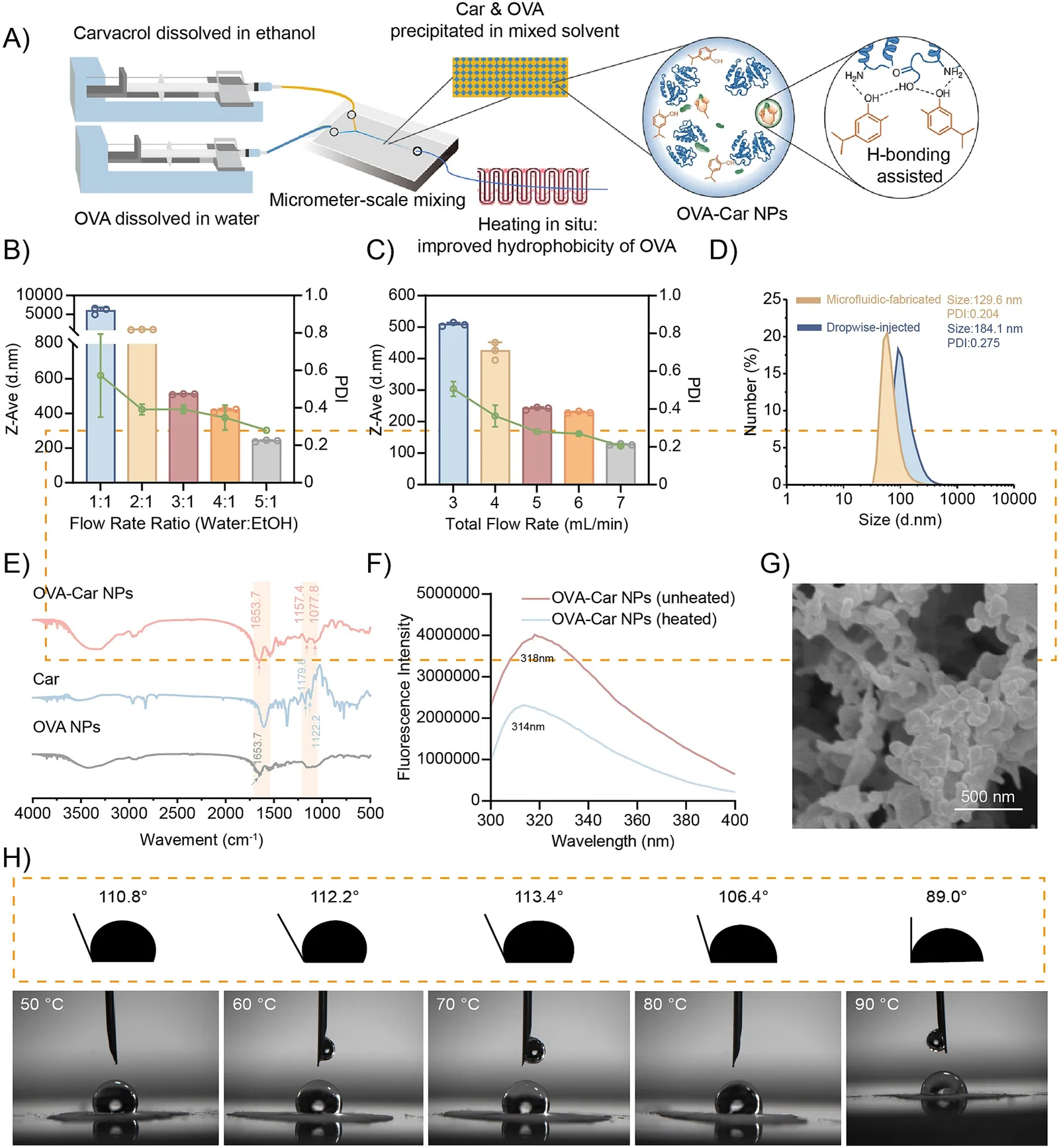
Characterization of OVA-Car NPs. (A) Schematic illustration of the nanoparticle preparation process; (B) Optimization of the aqueous-to-organic phase flow rate ratio (OVA solution-to-Car solution) and (C) total flow rate in the microfluidic system; (D) Size distribution of OVA-Car NPs; (E) FTIR spectra; (F) Intrinsic fluorescence spectra; (G) SEM micrographs; (H) Contact angles of OVA-Car NPs at different heating temperatures.

Under optimal conditions (OVA-to-Car flow rate ratio = 5:1; total flow rate = 7 mL/min), the FTIR spectra of OVA NPs, Car, and OVA-Car NPs were acquired to characterize their chemical structures and intermolecular interactions (Fig. 1E). Car showed characteristic peaks at 1600–1700 cm^−1^ (aromatic C=C stretching) and 1000–1200 cm^−1^ (C-O stretching), while OVA NPs exhibited the typical amide I (C=O) band. OVA-Car NPs retained the characteristic absorption peaks of both pure components, where the intensified signals at 1000–1200 cm^−1^ validated successful Car encapsulation. Peak shifts and intensity variations relative to pure OVA NPs further confirmed the formation of intermolecular hydrogen bonding and hydrophobic interactions, demonstrating effective Car loading.

Fluorescence spectroscopy (Fig. 1F) was employed to characterize OVA conformational variations within OVA-Car NPs, as this technique sensitively reflects microenvironmental alterations of hydrophobic amino acid residues and secondary structural transitions of proteins [33]. Heated OVA-Car NPs showed a 4 nm blue shift (318 nm to 314 nm) relative to unheated OVA NPs, indicating thermal treatment triggered OVA structural reorganization, likely involving partial burial of solvent-exposed aromatic residues (e.g., tryptophan, tyrosine) into protein hydrophobic core [25], [29]. These findings demonstrate that thermal treatment remodels OVA-Car nanoparticles into a more compact and hydrophobic conformation, which is conducive to improved interfacial activity-a prerequisite for high-performance Pickering emulsifiers.

The morphological characteristics of OVA-Car nanoparticles were characterized via SEM imaging (Fig. 1G). OVA-Car NPs under optimal conditions showed homogeneous quasi-spherical morphology with moderately rough surface. Their dimensions fell within the nanoscale range (consistent with earlier particle size analysis), which is typical for protein-based nanoparticles prepared via anti-solvent precipitation. Notably, moderate surface roughness facilitates nanoparticle anchoring at oil-water interfaces, thereby strengthening Pickering emulsion stabilization.

Contact angle measurements evaluated surface wettability, a pivotal factor for emulsifying performance: θ > 90° favors water-in-oil (W/O) emulsions, θ < 90° favors oil-in-water (O/W) emulsions, and θ near 90° enables superior interfacial adsorption and barrier formation [34]. As illustrated in Fig. 1H, 90 °C heat treatment increased the OVA-Car NP contact angle to 89.0° (near the 90° critical value), indicating exposed hydrophobic groups and enhanced hydrophobicity [25], [28]. These results verify that thermally treated OVA-Car NPs possess desirable wettability for interfacial anchoring, enabling their application as high-performance Pickering emulsifiers. Thermal treatment therefore provides a facile strategy to modulate particle surface hydrophobicity and tailor emulsification performance.

To evaluate the structural stability of OVA-Car nanoparticles under ultrasonic treatment, the nanoparticles were subjected to the same sonication conditions (20 kHz, 300 W, 6 min) used for Pickering emulsion fabrication. As shown in Fig. S1, no significant changes were observed in hydrodynamic diameter (from 182.9± 7.0 nm to 183.5 ± 6.9 nm, *p* > 0.05), PDI (from 0.24 ± 0.04 to 0.24 ± 0.03), zeta potential (from -28.3 ± 0.6 mV to -28.4± 0.9 mV), or water contact angle (from 89.3 ± 1.2° to 90.7 ± 1.0°) after sonication. These results indicate that the OVA-Car nanoparticles possess sufficient mechanical stability to withstand acoustic shear forces during emulsification, which can be attributed to the robust intermolecular hydrogen bonding and hydrophobic interactions established within the nanoparticle matrix. The maintained particle size and surface properties ensure that the nanoparticles retain their Pickering functionality after ultrasonic emulsification.

### 3.2 Ultrasonic cavitation modulates the physicochemical performance of pickering emulsions

Optical microscopy (Fig. 2A-B) showed that both ultrasonic power (100–400 W) and duration (2–8 min) exerted a unimodal influence on droplet morphology: droplet size decreased and became most uniform at 300 W and 6 min, respectively, while excessive intensity induced moderate coarsening. This trend was corroborated by steady-state flow curves (Fig. 2C-D), where apparent viscosity peaked under the same optimal conditions before declining at higher intensities. The frequency sweep results (Fig. 2E-F) further demonstrated predominant elastic behavior (G′ > G″) with weak frequency dependence, confirming the formation of a gel-like network [27]. Both G′ and G″ reached their maxima at 300 W and 6 min (1850 Pa and 1800 Pa at 1 rad/s), consistent with the finest droplet size [31], [35]. Notably, while 400 W sonication reduced both viscoelastic moduli, prolonged treatment for 8 min increased G′ despite droplet coarsening (Fig. 2B). This phenomenon is likely attributable to ultrasound-triggered protein aggregation, which induces droplet bridging flocculation and strengthens the elastic network, albeit compromising interfacial homogeneity [23]. Such ultrasonically optimized densely packed interfacial architecture enables efficient co-encapsulation of OVA-Car and Lut with enhanced structural stability.

**Fig. 2:**
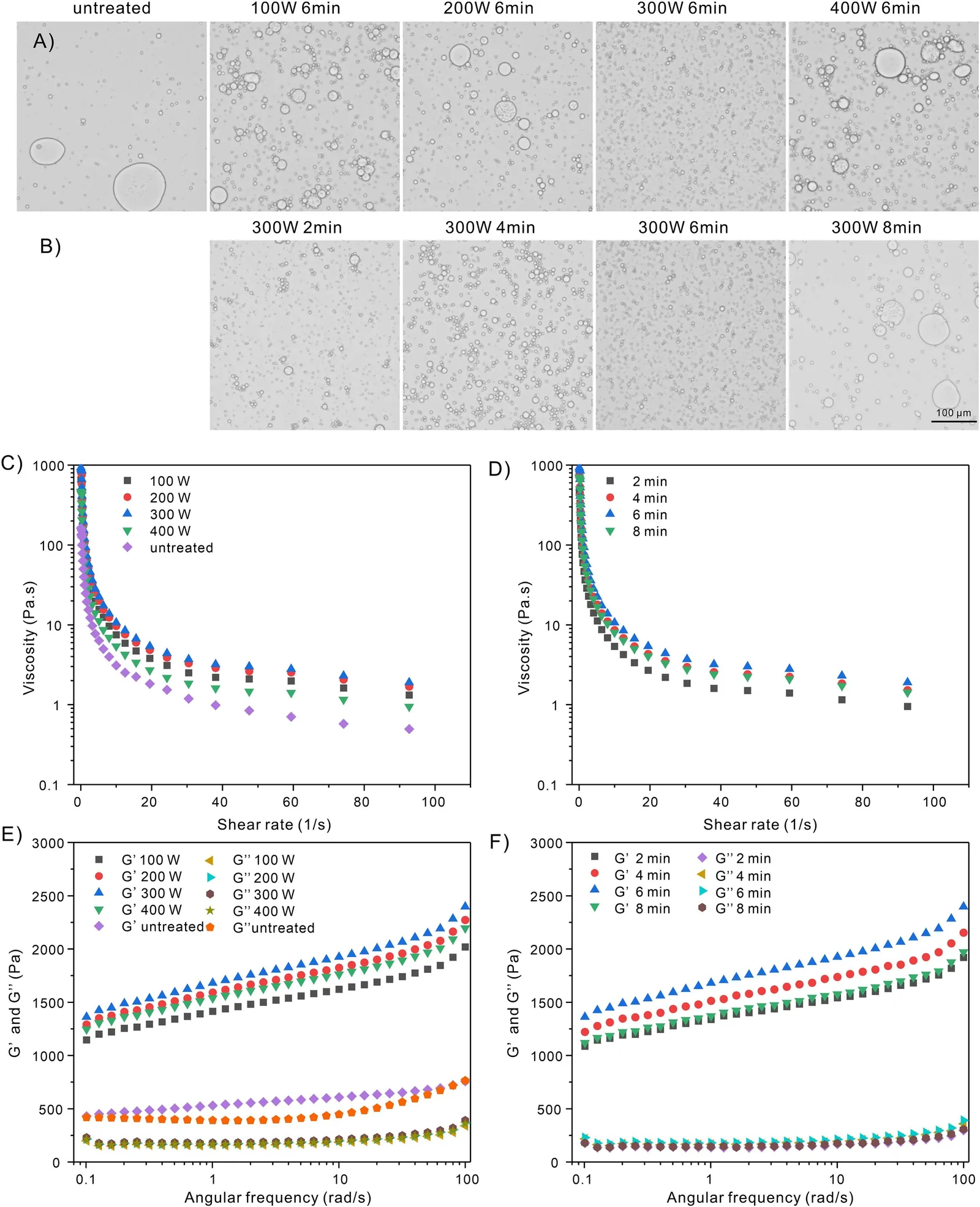
Effects of ultrasonic power and time on microstructure and rheological properties of OVA-Car Pickering emulsions. (A, B) Optical micrographs of emulsions with varying powers (fixed 6 min) and times (fixed 300 W), respectively; untreated emulsion as control. Scale bar = 100 μm. (C, D) Steady shear viscosity curves as a function of shear rate. (E, F) Frequency sweep curves.

As illustrated in Fig. 3A, the developed OVA-Car@Lut Pickering emulsion presents excellent injectability, satisfying the operational requirements for food surface coating. CLSM was employed to identify the emulsion type (Fig. 3B). FITC-labeled (green) OVA-Car nanoparticles were observed surrounding the oil droplets, while Nile Red-stained (red) corn oil formed the dispersed phase. The merged fluorescence images confirmed the oil-in-water (O/W) structure, with protein nanoparticles densely adsorbed at the oil–water interface as Pickering stabilizers. This O/W configuration is consistent with the hydrophilic nature of the OVA-Car nanoparticles (contact angle < 90°, as characterized in Section 2.2.2) and is favorable for aqueous-based edible coating applications.

**Fig. 3:**
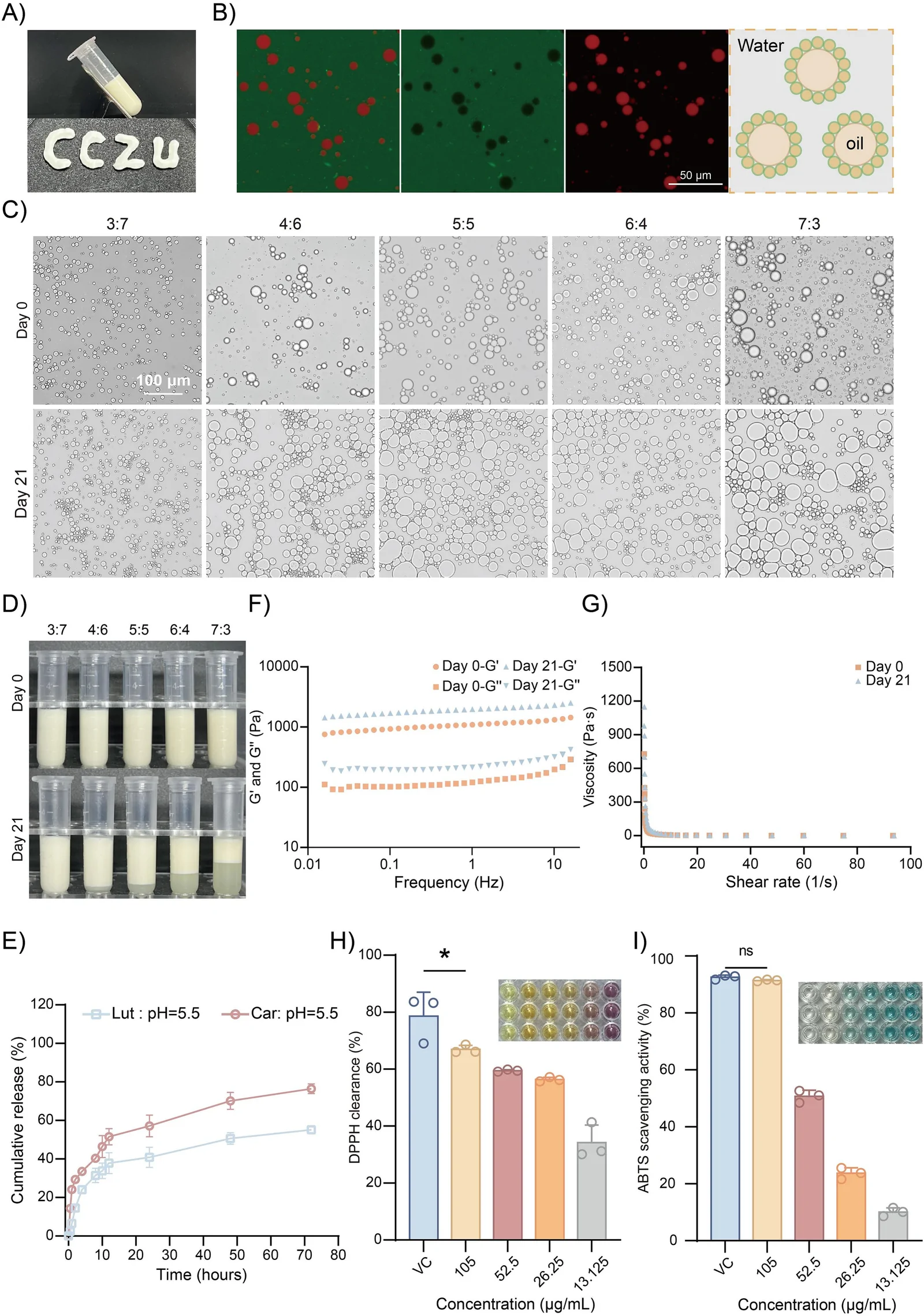
Characterization of OVA-Car@Lut Pickering emulsions. (A) Photographs of the emulsions; (B) CLSM images and emulsion type identification; (C) Optical micrographs, (D) macroscopic appearance, and (E) in vitro release profile; (F) Changes in storage modulus (G′) and loss modulus (G″) on day 0 and day 21 of storage; (G) Variation of apparent viscosity during 21-day storage; (H) DPPH and (I) ABTS Radical scavenging activities.

Optical microscopy (Fig. 3C) revealed that emulsion droplet size decreased and became more uniform as the aqueous-to-oil ratio increased from 3:7 to 7:3. The 7:3 (water/oil) group exhibited the smallest and most homogeneous droplets, which remained uniformly distributed after 21 days of storage, indicating superior long-term stability. Macroscopic stability assessment (Fig. 3D and Fig. S2) showed no visible stratification in any sample on day 0; after 21 days, only the 7:3 group retained a homogeneous appearance without phase separation or oiling-off, demonstrating strong coalescence resistance. This stability is attributable to the densely packed droplet arrangement at higher water fractions, which enhances inter-droplet steric repulsion and prevents gravitational creaming. Similar trends were observed in centrifugal and thermal stability tests (Fig. S3-S4).

In vitro release profiles at pH 5.5 (a food storage-relevant condition, Fig. 3E) showed both Lut and Car underwent rapid release within the first 10 h, followed by sustained slow release up to 80 h. Car exhibited higher cumulative release than Lut over 80 h, which can be ascribed to its lower hydrophobicity and weaker matrix interaction. This behavior confirms that the fabricated system achieves component-specific controlled release, enabling long-term preservation performance. (The calibration curves in Fig. S5-S6). Rheological analysis (Fig. 3F-G) showed the emulsion’s storage modulus (G′) was consistently higher than loss modulus (G″) on day 0 and 21, indicating a dominant elastic network structure (Fig. 3F); viscosity decreased with increasing shear rate, exhibiting typical pseudoplastic shear-thinning behavior (Fig. 3G).

Antioxidant capacity is a critical indicator for assessing the preservation performance of food-grade emulsions. The antioxidant performance of the developed Pickering emulsions was evaluated based on the synergistic effects of carvacrol and luteolin. As a natural flavonoid with prominent free radical‑scavenging activity, luteolin was incorporated into the oil phase and cooperated with carvacrol to substantially improve the overall oxidative stability of emulsion systems. [36], [37]. DPPH and ABTS assays (Fig. 3H-I) showed the emulsion achieved radical scavenging rates of 66.92 % ± 1.48 % (DPPH) and 91.40 % ± 0.29 % (ABTS) at 105 μg/mL Lut and 112.5 μg/mL Car. The sustained release profile of carvacrol from the Pickering emulsion further ensures prolonged antioxidant protection during postharvest storage [24].

### 3.3 Preparation and characterization of OVA-Car@Lut pickering films

Appearance and mechanical flexibility are critical for food packaging film applications. Fig. 4A illustrates the flexibility of the prepared films, including their deformation-related mechanical behavior under bending, folding, stretching, and twisting. The films exhibited a yellowish appearance, with visible light transmittance decreasing gradually with increasing Pickering emulsion content (Fig. 4B), indicating enhanced light-shielding capacity. UV-shielding performance also improved progressively with rising emulsion addition, peaking at 50 % emulsion content, with significantly superior UV-blocking capacity to pure chitosan films (Fig. 4E), consistent with previous reports [38]. Collectively, Pickering emulsion incorporation effectively enhanced film UV-blocking capacity, which helps delay light-induced food oxidation and degradation.

**Fig. 4:**
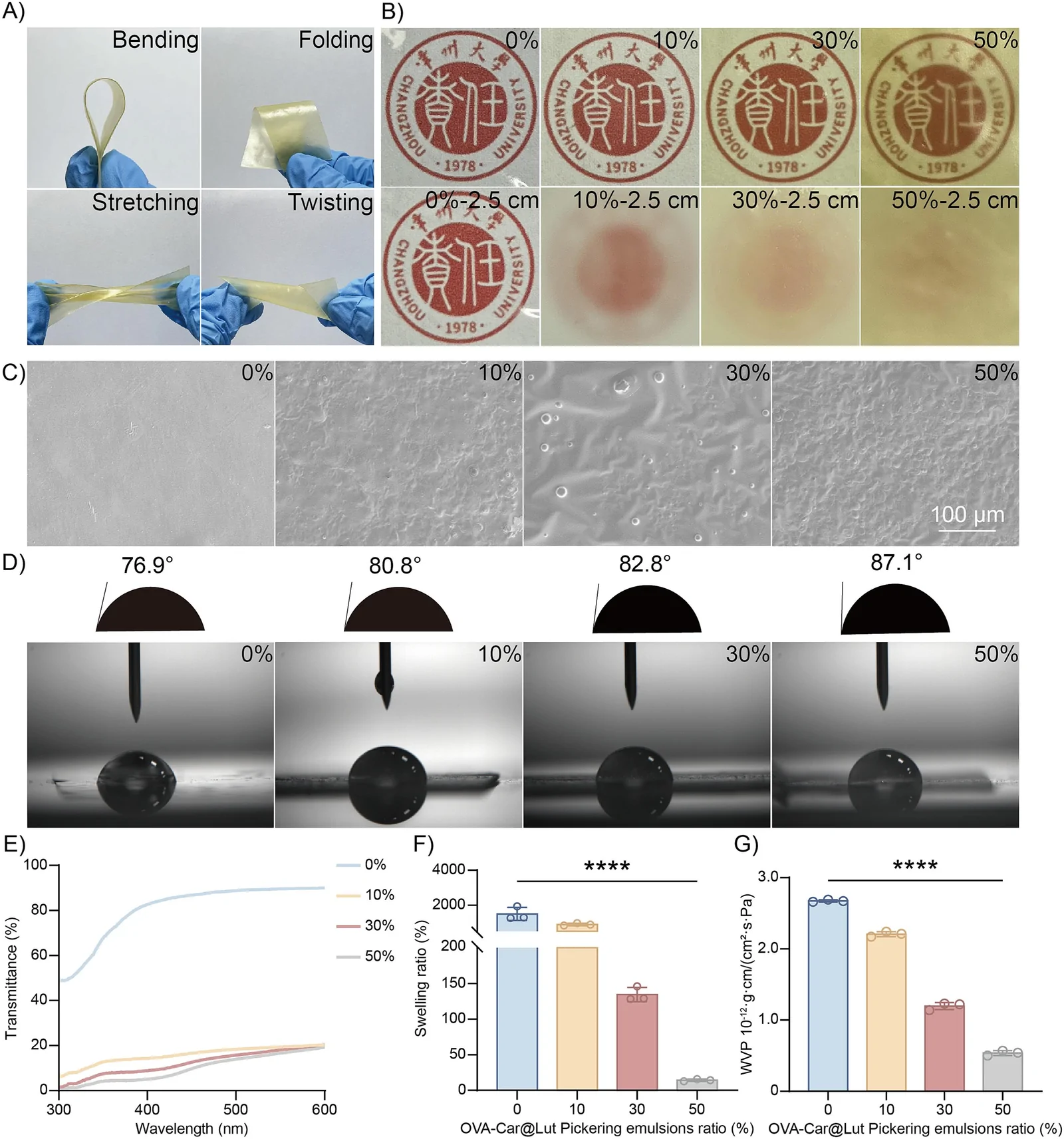
Characterization of OVA-Car@Lut Pickering emulsion-based films. (A) Digital images of OVA-Car@Lut Pickering emulsion-based films under twisting, bending, folding, and stretching tests; (B) Appearance of films incorporated with different amounts of Pickering emulsion; (C) SEM micrographs of the film; (D) Contact angles of the film; (E) UV absorption spectra in the wavelength range of 200–600 nm; (F) Swelling ratio; (G) WVP.

SEM images (Fig. 4C) present chitosan-based films with different Pickering emulsion incorporation ratios (0 %, 10 %, 30 %, and 50 %). The pure chitosan film (0 %) exhibited a smooth and uniform surface. With increasing emulsion incorporation (10 % to 50 %), film surfaces became gradually rougher with increased particulate structures, yet all films maintained continuous, structurally intact surfaces due to good component compatibility. When the Pickering emulsion content reached 50 %, the film surface displayed a uniform and dense particle distribution without noticeable aggregation or cracks. This morphology indicates thorough co-assembly between the Pickering emulsion and chitosan matrix at high emulsion ratios, where interfacial interactions synergistically reinforced the structure, optimizing the microstructure. Such a surface not only provides abundant loading sites for active components but also enhances interfacial performance through particle synergy, thereby furnishing a critical structural basis for the excellent preservation function of the films.

Contact angle measurements (Fig. 4D) showed the 50 % emulsion composite film had an increased contact angle from 78.9° to 87.1° compared with the pure chitosan film, indicating enhanced surface hydrophobicity. This demonstrates that emulsion incorporation effectively modulates film surface wettability and improves hydrophobicity. Previous studies have demonstrated that the addition of essential oil nanoemulsions can induce molecular rearrangement within the polymer matrix; concurrently, the nanoemulsions may enhance hydrophobic interactions with chitosan and promote the exposure of hydrophobic functional groups at the film surface, collectively contributing to the observed increase in film hydrophobicity [39]. Therefore, the observed increase in the contact angle of the composite films can be directly attributed to the hydrophobic nature of the incorporated emulsion.

Water vapor permeability (WVP), a key indicator for evaluating the critical moisture barrier properties of food packaging, decreased significantly from 2.21 ± 0.04 to 0.54 ± 0.04 (g·mm)/(m^2^·d·kPa) as emulsion content rose from 10 % to 50 % (Fig. 4G). The water vapor barrier property is strongly governed by film microstructure, surface wettability balance, and structural compactness. The improved barrier performance originates from the encapsulated lipophilic Car and Lut, which form internal hydrophobic barriers and suppress water molecule diffusion within the film matrix. At the same time, the incorporation of the emulsion enhanced intermolecular interactions among polymer chains, resulting in a denser film structure and thereby markedly improving moisture barrier performance. Similar findings have been reported in previous studies [40], where the incorporation of Pickering emulsions into chitosan (CS)-based composite films significantly enhanced their moisture resistance.

Fig. 4F further illustrates the effect of varying emulsion incorporation levels on the swelling ratio of the films. The swelling ratio reflects the dynamic penetration and diffusion of water within the films, and it is a key indicator for evaluating the hydrophilic properties of the films [41]. As the proportion of emulsion increased, the swelling ratio of the films gradually decreased, with a significant reduction observed when the emulsion content reached 50 %, where the swelling ratio dropped to 14.28 % ± 1.61. This reduction can be attributed to the hydrophobic characteristics of Car and Lut within the emulsion, which inhibited the penetration of water molecules into the films and consequently reduced their swelling capacity [24], [42].

Based on the comprehensive evaluation of mechanical flexibility, UV-shielding performance, water vapor permeability, and swelling ratio across the four Pickering emulsion incorporation ratios, the 50 % formulation exhibited the optimal overall performance. Specifically, the 50 % emulsion film demonstrated the highest UV-blocking efficiency, the lowest water vapor permeability, and the lowest swelling ratio, while maintaining favorable mechanical flexibility for practical handling. Notably, the contact angle of the 50 % film reached 87.1°, which is already close to the 90° threshold for effective hydrophobic surfaces, and further increasing the emulsion content would offer only marginal improvement in surface wettability. Meanwhile, the light transmittance in the visible region (300–600 nm) showed negligible change when the emulsion content increased from 30 % to 50 %, indicating that the UV-shielding capability had essentially reached a plateau. Therefore, the 50 % Pickering emulsion formulation was selected as the optimal composition for the subsequent passion fruit coating experiments.

### 3.4 Antimicrobial activity

According to previous studies, both Car and Lut exhibit excellent antimicrobial activity [43], [44]. Nevertheless, the antimicrobial performance of Lut-loaded, OVA-Car-stabilized Pickering emulsions remains poorly understood. This study further evaluates the antibacterial and antifungal activities of the developed emulsions against three typical microbial strains via the plate coating method. As shown in Fig. 5A-D, the emulsions exhibited strong inhibitory effects, with inhibition rates of ≥75 % for all three strains. Among them, the most pronounced effect was observed against *S. aureus*, followed by *E. coli* and *A. niger*.

**Fig. 5:**
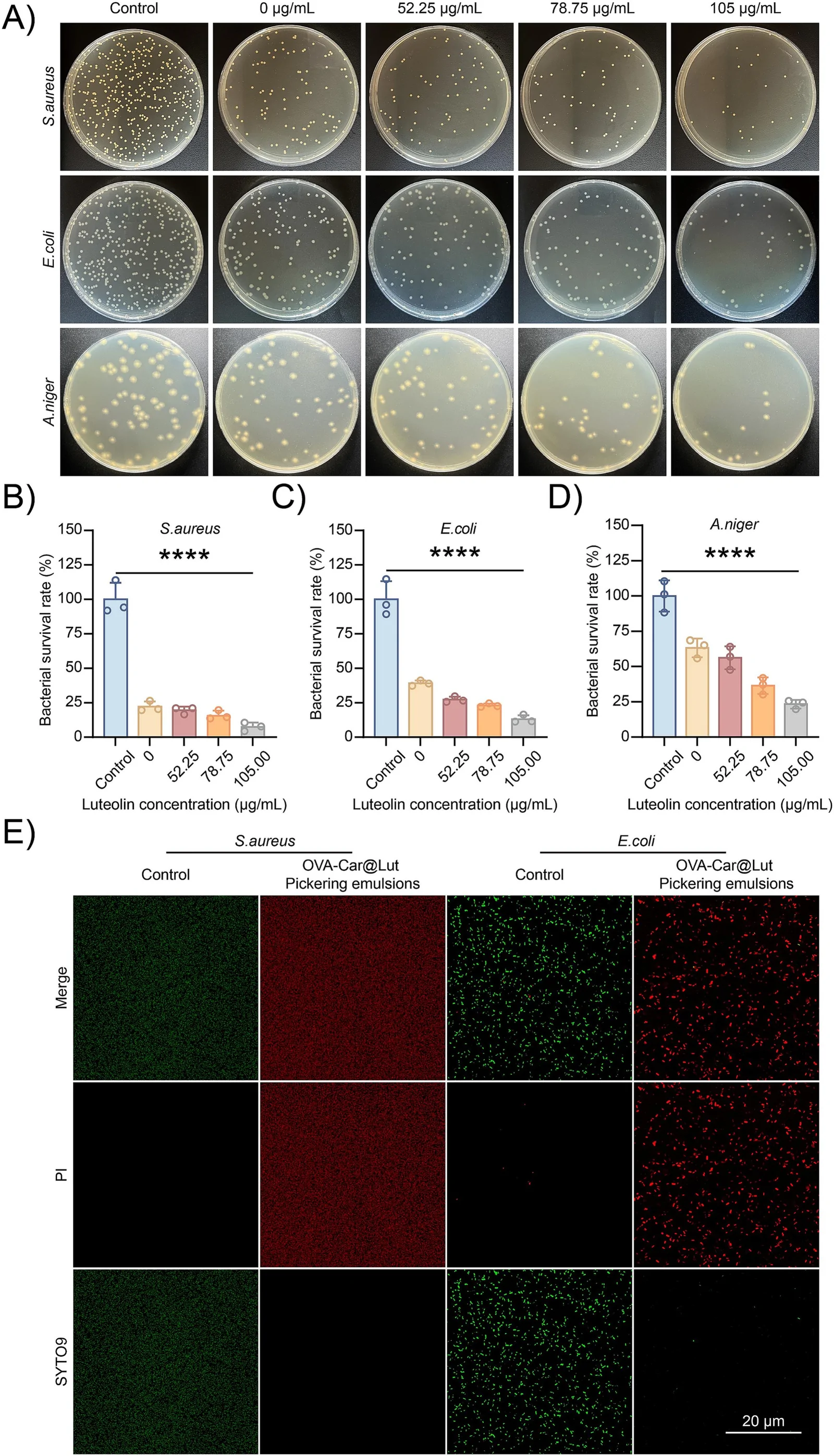
Antibacterial properties of OVA-Car@Lut Pickering emulsions. (A) Plate counting results of OVA-Car@Lut Pickering emulsions against *S. aureus*, *E. coli* and *A.niger*; Inhibition rates of the OVA-Car@Lut Pickering emulsion against *S. aureus* (B), *E. coli* (C) and *A. niger* (D); (E) Live/dead staining of *S. aureus* and *E. coli*.

Propidium iodide (PI) stains dead bacterial cells, while SYTO9 stains viable ones. PI-stained cells emit red fluorescence, whereas SYTO9-stained cells show green fluorescence. Fig. 5E shows negligible red fluorescence in the untreated group, indicating mostly viable cells. In contrast, emulsion-treated bacteria exhibited predominantly red fluorescence with minimal green fluorescence, suggesting extensive cell death. This rise in dead bacteria links to biofilm disruption, compromising structural integrity and reducing stress resistance.

### 3.5 Antimicrobial mechanism

Fig. 6B illustrates morphological differences between untreated and emulsion-treated microbes. Untreated *S. aureus* had normal coccoid shape with smooth surfaces. In contrast, after emulsion treatment, the bacterial morphology was markedly altered, with cells displaying distorted shapes and loss of structural integrity [45]. Similarly, *E. coli* appeared as intact, rod-shaped cells prior to treatment, whereas treated cells exhibited significant morphological changes, becoming irregular and shrunken [46]. The morphology of *A. niger* showed distinct differences before and after treatment. In the untreated group, the hyphal structures were uniform, with smooth and compact surfaces, without visible damage. After treatment with the emulsion, the hyphae exhibited pronounced shrinkage and collapse, indicating possible disruption of the cell membrane integrity, which may have resulted in growth inhibition or cell death of *A. niger*. These distinct morphological damages confirm that the Pickering emulsion disrupts bacterial and fungal cell membrane integrity, triggering membrane rupture and intracellular substance leakage. The distorted cellular architecture further increases membrane instability and accelerates cytoplasmic efflux. Fig. S7-S8 illustrate the emulsion’s disruption and inhibition of *S. aureus* and *E. coli* biofilms. Results indicate that the emulsion exerted pronounced biofilm disruption and inhibition against both bacterial species, with particularly notable effects on *S. aureus* biofilms.

**Fig. 6:**
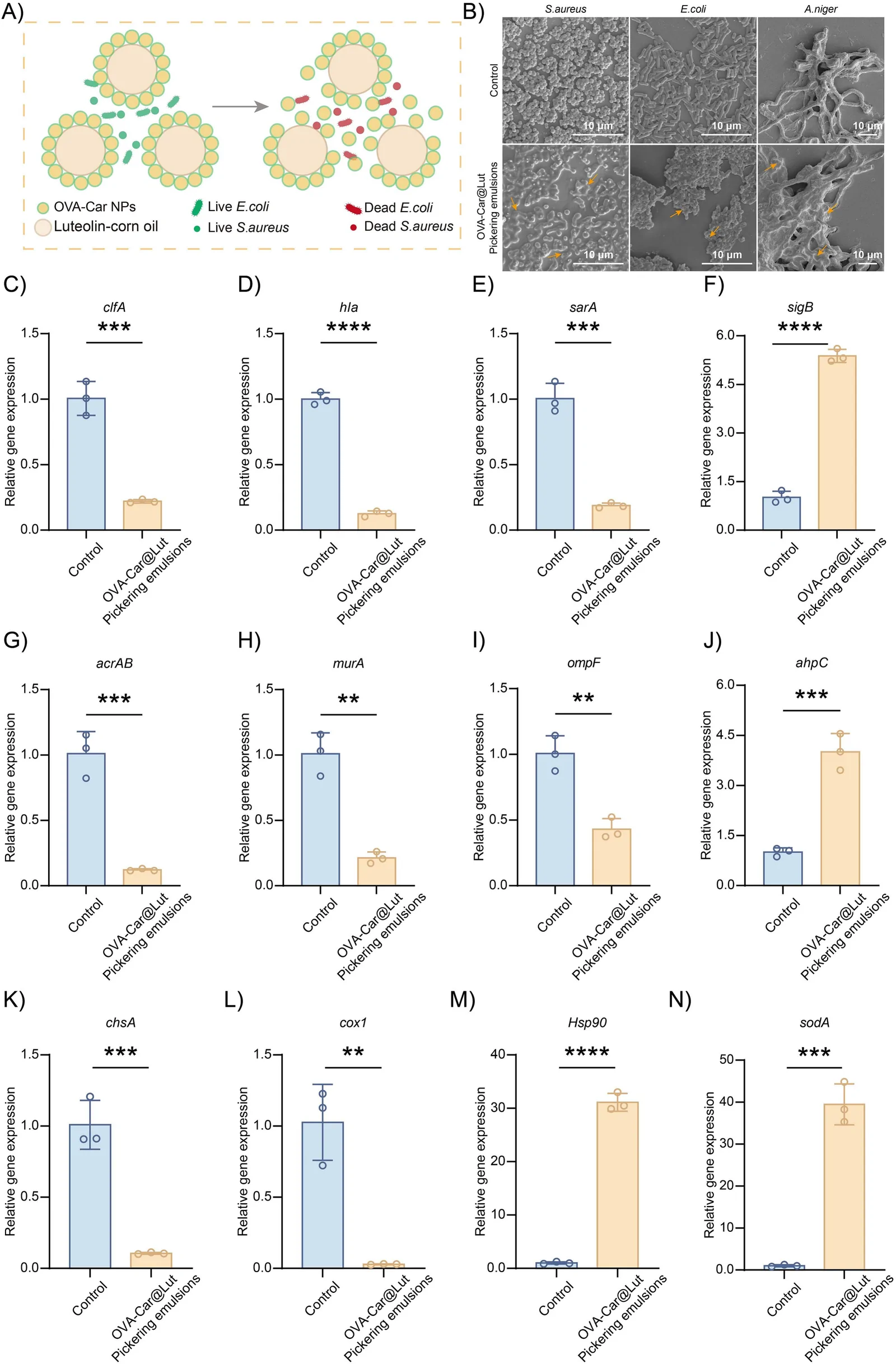
Antibacterial mechanism of OVA-Car@Lut Pickering emulsions. (A) Schematic representation of the proposed antibacterial mechanism; (B) SEM images of untreated and Pickering emulsion-treated *S. aureus*, *E. coli* and *A. niger*. Scale bar represents 10 μm. RT-qPCR analysis of gene expression in (C-F) *S. aureus*, (G-J) *E. coli*, and (K-N) *A. niger* treated with OVA-Car@Lut Pickering emulsions.

To further elucidate the emulsion’s antibacterial mechanism against the three microbes, we analyzed genes related to stress response, cell wall synthesis, and biofilm formation. Relative gene expression levels of treated and untreated strains are shown in Fig.6 C-J. For *S. aureus*, the expression of four key genes (*clfA, hla, sarA, sigB*) was altered: *clfA, hla, and sarA* were downregulated, while *sigB* was significantly upregulated. *clfA/hla/sarA* downregulation may compromise virulence and adhesion, attenuating pathogenicity; *sigB* upregulation reflects adaptive response to emulsion-induced stress [47]. Collectively, these results suggest that OVA-Car@Lut Pickering emulsions may exert antibacterial activity by suppressing virulence-associated gene expression and modulating bacterial stress response pathways at the transcriptional level. For *E. coli*, four key genes (*acrAB*, *murA*, *ompF*, *ahpC*) were investigated: *acrAB*, *murA*, and *ompF* were significantly downregulated, and *ahpC* was markedly upregulated. *acrAB* (efflux pump) downregulation may reduce drug resistance; *murA* (cell wall synthesis) downregulation impairs integrity; *ompF* (outer membrane porin) downregulation limits drug uptake. *ahpC* upregulation enhances antioxidant defense against oxidative stress. Therefore, it is plausible that the emulsion may attenuate E. coli resistance and compromise cellular viability through transcriptional modulation of genes involved in efflux pump activity, cell wall biosynthesis, outer membrane permeability, and oxidative stress responses. However, these inferences are primarily derived from mRNA expression changes and require further validation at the protein and functional levels.

For *A. niger*, *hsp90* and *sodA* were upregulated; *cox1* and *chsA* were significantly downregulated (Fig. 6K-N). *hsp90* upregulation (a membrane damage marker) indicates disrupted integrity; *chsA* downregulation impairs chitin synthesis, inhibiting cell wall biosynthesis. *sodA* upregulation reflects ROS accumulation and activated antioxidant defense; *cox1* downregulation suppresses mitochondrial respiration and energy metabolism. Overall, the transcriptional changes observed in *A. niger* suggest that the Pickering emulsion may interfere with membrane integrity, antioxidant defense, and energy metabolism pathways. These findings provide preliminary molecular clues for the antifungal mechanism, but further biochemical assays are needed to confirm the proposed pathways.

### 3.6 The application of preserving passion fruit

To evaluate the practical preservation performance of OVA-Car@Lut Pickering coatings, passion fruit surfaces were coated via a dipping approach, and shelf-life extension effects were systematically investigated. As illustrated in the experimental timeline (Fig. 7A), preservation treatments started on day 0, with tissue samples collected on days 0, 3, 5, and 7 to measure relevant physicochemical changes, providing a comprehensive evaluation of the coating’s preservation performance.

**Fig. 7:**
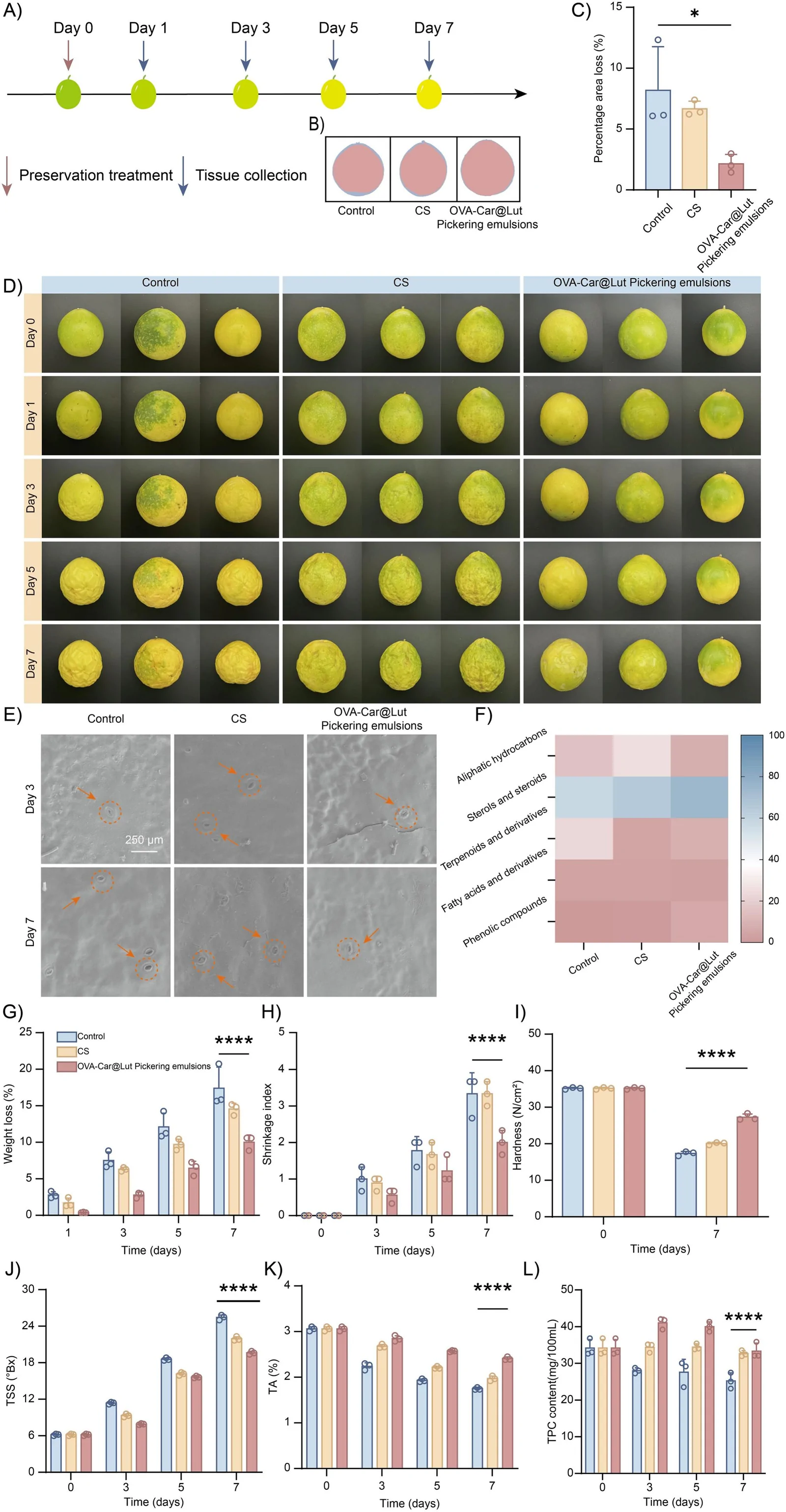
(A) Ilustration of experimental design and timeline. (B) Shrinkage area and (C) corresponding quantitative analysis; (D) Photographs of passion fruit during storage; (E) SEM micrographs of passion fruit peel; (F) GC-MS analysis; Changes in (G) weight loss, (H) shrinkage index, (I) titratable acidity (TA), (J) total soluble solids (TSS), (K) hardness, and (L) total phenolic content (TPC) of passion fruit during storage.

Representative images from each group were compiled into stacked images of passion fruits (Fig. 7B), and the corresponding areas were quantified (Fig. 7C). Quantitative analysis showed that the coating-treated group had the lowest area reduction, indicating superior water-loss control. As a climacteric fruit, passion fruit rapidly consumes nutrients post-harvest due to high respiration and transpiration, leading to dehydration and short shelf life [48]. Therefore, the weight loss behavior of untreated and coated fruits at room temperature was compared (Fig. 7G). Specifically, by day 7, the weight loss of the coated fruits was restricted to 10.2 %, compared to a much higher loss of 17.4 % in the uncoated control group. These results verify that the Pickering emulsion coating efficiently alleviates postharvest weight loss. This beneficial effect is attributed to the coating’s semi-permeable barrier behavior toward O_2_, CO_2_ and water vapor, which suppresses surface water evaporation and reduces mass loss during storage.

SEM observations of passion fruit peels (Fig. 7E) revealed that in both the control and CS-treated groups, stomata were only slightly open on day 3 but became fully open by day 7. In contrast, the Pickering emulsion-coated fruits maintained closed stomata even on day 7. These results further indicate that the Pickering emulsion coating effectively delays quality deterioration in passion fruit during storage. These comprehensive findings demonstrate that the Pickering emulsion coating maintains fruit firmness and moderates postharvest respiration, while its inherent antimicrobial activity suppresses microbial spoilage, thereby delaying overall quality deterioration.

To investigate changes in passion fruit volatile components during storage and the effect of preservation treatments, a GC-MS heatmap (Fig. 7F) illustrates volatile metabolite relative abundances in different groups after 7 days of storage. The heatmap revealed the coated group retained more key freshness-related metabolites than the CS and control groups, with the highest relative abundances of sterols/steroids (linked to membrane integrity) and terpenoids/derivatives (associated with aroma quality); the control group showed notable reductions in these components, while the CS group had intermediate preservation. Fatty acids and their derivatives remained relatively stable across treatments, indicating minimal impact on lipid metabolism; a slight increase in phenolics in the coated group may reflect enhanced retention of antioxidant-related constituents [49]. These variations are consistent with the typical protective mechanisms of emulsion-based edible coatings, which form semi-permeable barriers to mitigate moisture and gas exchange, restrain volatile component dissipation, and retard physiological senescence, thereby preserving fresh fruit quality and extending shelf life [24]. Overall, OVA-Car@Lut Pickering treatment effectively preserves antioxidant-related volatiles and mitigates excessive aroma component changes, demonstrating pronounced preservative effects (substance proportions are shown in Table S2).

As shown in Fig. 7H, fruit shrinkage index is critical for evaluating passion fruit quality, as it affects visual integrity and correlates closely with ripening and senescence [50]. The data indicate that the shrinkage index increased over the storage period for all three groups, consistent with the changes observed in the fruit appearance shown in Fig. 7D. The control group’s shrinkage index increased significantly from day 3, reaching 3.33 ± 0.58 by day 7; in contrast, the Pickering emulsion-coated group had a markedly lower shrinkage index (2.00 ± 0.33) than the control and CS groups. These results indicate the Pickering emulsion coating effectively inhibits fruit shrinkage and visual deterioration, demonstrating superior preservation performance and extending passion fruit shelf life.

Fruit firmness is a key parameter for assessing passion fruit maturity and quality. Fig. 7I presents firmness changes during 7 days of room temperature storage: all groups exhibited gradual firmness decline. After 7 days, the control group’s firmness dropped to 17.33 ± 0.57 N/cm^2^ (50.73 % reduction from day 0), the CS-treated group to 20.07 ± 0.32 N/cm^2^ (42.93 % reduction), while the Pickering emulsion-coated group retained higher firmness (27.27 ± 0.9 N/cm^2^) with only a 22.46 % reduction. Notably, by day 5, visible brown spots caused by microbial invasion appeared on the surfaces of the control and CS-treated fruits (Fig. 7D), which is a common postharvest pathology in passion fruit. In comparison, the surface of the Pickering emulsion-coated fruits remained largely intact.

Total soluble solids (TSS) of passion fruit were measured during storage (Fig. 7J). By day 3, TSS in the control group increased significantly to 11.37 ± 0.15 °Brix, exceeding that of the CS-treated (9.33 ± 0.25 °Brix) and coated (7.90 ± 0.10 °Brix) groups. Over 7 days, TSS continued to increase in all samples, reaching 25.43 ± 0.40 and 21.93 ± 0.32 °Brix in the control and CS-treated groups, respectively, while the coated group showed a lower TSS (19.63 ± 0.25 °Brix) with a reduced increase. This variation originates from natural postharvest ripening and continuous water loss, which concentrates internal soluble sugar substances. Both CS and Pickering emulsion coatings limited water loss and slowed TSS increase, with the latter showing superior preservation performance and extended shelf life. These results agree with previous reports on edible composite coatings reducing TSS increases in yellow passion fruit [51].

Titratable acidity (TA) of the control group decreased from 3.06 ± 0.05 % (day 0) to 1.75 ± 0.02 % (day 7) (Fig. 7K). The TA of the CS-coated and Pickering emulsion-coated groups declined from an initial value of 3.06 ± 0.05 % (day 0) to 1.97 ± 0.05 % and 2.41 ± 0.04 % (day 7), respectively. The control group showed the most pronounced TA decline. TA is closely related to the organic acid content in fruits and serves as an indicator of changes in intrinsic fruit acidity over time [9], The reduction in acidity is a common feature of fruit ripening, associated with the consumption and metabolic transformation of organic acids [52]. In contrast, the Pickering emulsion coating effectively slowed TA decline, maintaining higher acidity and delaying ripening to preserve freshness. The coating forms an efficient barrier against gas exchange and water loss, enhancing preservation. Although the CS coating had some protective effect, its TA retention was not significantly different from the control, suggesting the Pickering emulsion coating—particularly encapsulated Car and Lut-plays a critical role in maintaining fruit acidity. Similarly, passion fruits treated with the Pickering emulsion group exhibited a smaller decrease in pH and retained the highest vitamin C content (Fig. S9-S11).

Phenolic compounds are secondary metabolites and key constituents that contribute to fruit resistance against oxidative stress and pathogenic attacks [53]. Total phenolic content (TPC) of passion fruit gradually declined during storage (Fig. 7L), primarily due to accelerated respiratory metabolism and oxygen exposure promoting phenolic oxidation/degradation. Compared with the control and CS-coated groups, the Pickering emulsion-coated group showed a markedly slower TPC decline. This is attributed to the coating forming a dense surface barrier, reducing oxygen diffusion and water loss to inhibit phenolic oxidation. Additionally, encapsulated natural antioxidants (Car and Lut) in the Pickering emulsion may mitigate phenolic degradation, effectively preserving TPC during storage.

## 4 Conclusion

This work established a novel microfluidic-ultrasonic coupled processing strategy for the fabrication of Pickering emulsion-based edible coatings targeted at passion fruit postharvest preservation. Microfluidic precision synthesis enables the fabrication of homogeneous OVA-Car@Lut nanoparticles with narrow size distribution and superior batch reproducibility. Subsequent ultrasonic emulsification was systematically optimized (100–400 W, 2–8 min), revealing that moderate cavitation (300 W, 6 min) optimally refined droplet size and maximized viscoelastic moduli, while excessive power induced structural deterioration. Notably, prolonged sonication (8 min) promoted bridging flocculation, reinforcing the elastic network at the expense of interfacial uniformity. The optimally ultrasonicated emulsions exhibited outstanding stability, sustained release, and synergistic antioxidant/antimicrobial activities (DPPH: 66.92 %, ABTS: 91.40 %, ≥75 % bacterial inhibition). When applied as chitosan-based coatings, the system restricted fruit weight loss to 10.2 % (vs. 17.4 % in controls) and extended shelf life to 7 days. This study verifies that the integration of microfluidic prefabrication and ultrasonic emulsification provides a scalable sonochemical platform for advanced postharvest preservation. Meanwhile, it offers valuable insights into ultrasound-mediated interfacial regulation of biopolymer-stabilized Pickering systems.

## CRediT authorship contribution statement

**Song-Wei Lv:** Writing – review & editing, Project administration, Investigation, Funding acquisition, Conceptualization. **Zhu-Huan Tang:** Writing – original draft, Methodology, Investigation, Formal analysis. **Zhi-Fan Ye:** Investigation. **Yi-Jing Lu:** Validation, Investigation. **Gao-Xiang Jin:** Validation, Investigation. **He-Ling Yu:** Visualization. **Da-Na Liu:** Writing – review & editing, Investigation. **Jianhao Wang:** Writing – review & editing, Investigation, Conceptualization.

## Funding

The research was funded by the National Natural Science Foundation of China (22204009), China Postdoctoral Science Foundation (2024M760298).

## Declaration of competing interest

The authors declare that they have no known competing financial interests or personal relationships that could have appeared to influence the work reported in this paper.

## Appendix A Supplementary data

Supplementary Data 1 This supplementary material provides detailed characterization of OVA-Car nanoparticles, stability assessments of OVA-Car@Lut Pickering emulsions, standard curves, antimicrobial/antibiofilm activities, passion fruit preservation data (pH, vitamin C, GC-MS), and primer sequences.
