# Supplementary material for: Microfluidics-engineered ovalbumin-carvacrol nanoparticles for ultrasonically fabricated pickering emulsion coatings: a sonochemical strategy for fruit preservation
Source: Ultrason Sonochem. 2026 Sep 23;133:108073. doi: 10.1016/j.ultsonch.2026.108073 (PMC13635944; doi:10.1016/j.ultsonch.2026.108073)
Supplement: Supplementary file 1 [file mmc1.docx]

**Supplementary Material**

**Figure S1**. Stability of OVA-Car nanoparticles before and after ultrasonic treatment (20 kHz, 300 W, 6 min). (A) Hydrodynamic diameter distribution measured by DLS; (B) Zeta potential; (C) Water contact angle. Data are presented as mean ± SD (n = 3).

**Figure S2**. Quantitative analysis of particle size variation related to storage stability of OVA-Car@Lut Pickering emulsions.

**Figure S3**. Centrifugal stability of OVA–Car@Lut Pickering emulsions: A) Inverted microscopy images; B) Digital photographs of emulsions; C) Quantitative analysis of particle size variation before and after centrifugation.

**Figure S4**. Heating stability of OVA–Car@Lut Pickering emulsions: A) Inverted microscopy images; B) Digital photographs of emulsions; C) Quantitative analysis of particle size variation before and after heating.

**Figure S5**. Standard curve of carvacrol.

**Figure S6**. Standard curve of luteolin.

**Figure S7**. Disruptive effects of OVA-Car@Lut Pickering emulsions on *S. aureus* and *E. coli* biofilms.

**Figure S8**. Inhibitory effects of OVA-Car@Lut Pickering emulsions on *S. aureus* and *E. coli* biofilms.

**Figure S9**. Vitamin C content of passion fruits in different experimental groups.

**Figure S10**. pH of passion fruits in different experimental groups.

**Figure S11**. Standard curve of vitamin C

**Table S1 Primer sequences**

| Strain of microorganism | Primer name | Primer sequence |
| --- | --- | --- |
| *S. aureus* | *sarA-F* | TGACATCAAGACTGCTTGGT |
|  | *SarA-R* | TGCATTGATGGTTTGTCTGC |
|  | *ClfA-F* | ACAGCAGGTGGTAGTGGTAA |
|  | *ClfA-R* | TTGCAGGTTTCGTTGCTGAT |
|  | *Hla-F* | GGTGCAAGTACAGCTGGTAA |
|  | *Hla-R* | CAGCTTTCGTTGCTGGTATT |
|  | *SigB-F* | CGACAAGATCAAGCACGTTT |
|  | *SigB-R* | TTGATCGTGTCGATTCCTGT |
|  | *16S Rrna-F* | GCTAATACCGCATACGTCCT |
|  | *16S Rrna-R* | GCTTCTCGTTCGATGTTAGG |
| *E. coli* | *OmpF-F* | GGTGTTCCGTACTTCAGCAA |
|  | *OmpF-R* | GTTGCGGTTCACTTTCTGAC |
|  | *AcrA-F* | GCTGGTGACTATCTGGCTGT |
|  | *AcrA-R* | GATGTTGCCGTAGATGTTGC |
|  | *AcrB-F* | TACAGCGTTCGCGCTGACAT |
|  | *AcrB-R* | CACGACGATACCCAGTTGTT |
|  | *MurA-F* | TGCGTGAACGTAAAACTGGA |
|  | *MurA-R* | GTTGATCAGCCGATAGGTGA |
|  | *AhpC-F* | GCTGGTTTCGACGTTGAAAT |
|  | *AhpC-R* | ATGCACACCAGACTTCACCA |
|  | *16S Rrna-F* | GTGCCAGCAGCCGCGGTAA |
|  | *16S Rrna-R* | GGACTACCAGGGTATCTAAT |
| *A.niger* | *ChsA-F* | GCGGTCAACTACATGGACTT |
|  | *ChsA-R* | TCAGCACCTTGGTGTAGATG |
|  | *Soda-F* | ACGACGGTAAAGCTGACTCT |
|  | *Soda-R* | GATGTTGCCGATCTGGTTGA |
|  | *Hsp90-F* | CGTCTTCGAGTACCTGGAGA |
|  | *Hsp90-R* | GACGATGTTGCCGATCTTGA |
|  | *cox1-F* | GGATTTGGTAACCTAGCAGGC |
|  | *cox1-R* | GCATAGTTGGTCCGATAAGC |
|  | *ActA-F* | GTGCCCATCTACGAGGGTTA |
|  | *ActA-R* | GCAGGTGTCAGTGATGGTGA |

**Table S2 GC–MS quality analysis of passion fruit pulp on the 7th day of storage**

| Type of Compound Name | Control | CS | OVA-Car@Lut Pickering emulsions |
| --- | --- | --- | --- |
| Aliphatic hydrocarbons | 14.903% | 25.734% | 7.75% |
| Sterols and steroids | 58.078% | 65.371% | 74.836% |
| Terpenoids and derivatives | 22.639% | 4.413% | 8.615% |
| Fatty acids and derivatives | 3.766% | 3.49% | 3.194% |
| Phenolic compounds | 0.615% | 0.991% | 5.606% |
